# The GnRH Agonist Triptorelin Causes Reversible, Focal, and Partial Testicular Atrophy in Rats, Maintaining Sperm Production

**DOI:** 10.3390/ijms26146566

**Published:** 2025-07-08

**Authors:** Alberto Marcos, Maria Cruz Rodríguez del Cerro, Rosa María Fernández, Eduardo Pásaro, Nuria Arias-Ramos, Pilar López-Larrubia, Pilar González-Peramato, Antonio Guillamon, Maria P. De Miguel

**Affiliations:** 1 Department of Psychobiology, Universidad Nacional de Educación a Distancia, 28040 Madrid, Spain; albmarcos@pas.uned.es (A.M.); mcrdelcerro@psi.uned.es (M.C.R.d.C.); aguillamon@psi.uned.es (A.G.); 2 Department of Psychology, Interdisciplinary Center for Chemistry and Biology Institute (CICA), DICOMOSA Group, University of A Coruña, 15190 A Coruña, Spain; rosa.fernandez@udc.es (R.M.F.); eduardo.pasaro@udc.es (E.P.); 3 Department of Psychology, Institute for Biomedical Research of A Coruña (INIBIC), 15006 A Coruña, Spain; 4 Biomedical Magnetic Resonance, Instituto de Investigaciones Biomédicas Sols-Morreale, CSIC-UAM, 28029 Madrid, Spain; narias@iib.uam.es (N.A.-R.); plopez@iib.uam.es (P.L.-L.); 5 Biomedical Research Networking Centre on Rare Diseases (CIBERER), Institute of Health Carlos III, 28029 Madrid, Spain; 6 Cell Engineering Laboratory, La Paz University Hospital Health Research Institute (IdiPAZ), 28046 Madrid, Spain; mpilar.gonzalezperamato@salud.madrid.org; 7 Department of Pathology, La Paz University Hospital, Universidad Autónoma de Madrid, 28046 Madrid, Spain

**Keywords:** GnRH agonists, triptorelin, rat testes, fertility, transgender girls

## Abstract

We aim to provide a translational model to investigate the reproductive consequences of pubertal delay using the GnRH agonist triptorelin in transgender girls, tested in particular on testicular maturation in peripubertal rats. A total of 30 Sprague Dawley rats were utilized, with 10 subjects assigned to each of three groups from day P30 postpartum (prepubertal) until day P95 (postpubertal), mimicking treatment timing in patients. Rats received triptorelin at three time points (P30, P50, and P71), or only at P30 and P50. Control rats were injected with vehicle. Plasma testosterone levels were determined using MRM analysis. Testes and epididymides were examined histologically. There were significantly lower testosterone levels at postnatal day 48 in treated rats, indicating delayed puberty, with further reductions by day 69. By day 93, testosterone levels had recovered in rats given vehicle at P71 but remained low in the triptorelin-continuous group, suggesting the reversibility of the treatment. Treated rats had smaller testes; however, the majority of the testicular parenchyma was unaffected, with most seminiferous tubules displaying complete spermatogenesis. However, focal atrophic changes were observed in 1–30% of the parenchyma. One-third of the short-term group and half of the long-term group were classified as atrophic. Despite these changes, all treated rats had mature sperm in the epididymis, ensuring their fertility. In conclusion, triptorelin treatment promotes a decline in testosterone levels accompanied by discrete atrophy of the seminiferous tubules, which is partially reversible and compatible with sperm production and fertility preservation. Triptorelin could be an appropriate treatment prior to estrogen therapy for patients seeking gender transition.

## 1. Introduction

Gender identity is one’s consciousness of being a man, woman [1], or other condition. A definition from the American Psychological Association [2] states that gender identity is “…a person’s deeply-felt, inherent sense of being a boy, a man, or a male; a girl, a woman, or a female; or an alternative gender (e.g., genderqueer, gender nonconforming, gender neutral) that may or may not correspond to a person’s sex assigned at birth or to a person’s primary or secondary sex characteristics” [2].

Reliable statistics on the prevalence of transgender individuals are limited, as not all transgender people seek hormonal or surgical affirmation treatment through healthcare services. The Diagnostic and Statistical Manual of Mental Disorders, fifth edition, (DSM-5) estimates a prevalence of 0.002% to 0.014% in adults [3], while the Williams Institute estimates 0.6% for those aged 13 and older [4]. In Europe, the reported prevalence is 4.6 per 100,000 individuals [5]. Among children and adolescents, estimates range from 1.2% to 2.7% [6].

Some transgender children and adolescents experience gender incongruence (GI) according both the International Classification of Diseases 11th Revision (ICD-11) and gender dysphoria (GD) (DSM-5-TR) [7]. GI refers to cognitive aspects, while GD addresses the emotional aspects. GI describes a person’s experience of incompatibility between their gender identity and the gender assigned at birth [8], whereas GD refers to the distress that may accompany this incongruence [7].

Transgender children experiencing GD who reach Tanner stages 2–3 or later may be prescribed gonadotropin-releasing hormone agonists (GnRHa), such as triptorelin. As a GnRH agonist, it initially stimulates and then desensitizes pituitary GnRH receptors, reducing gonadotropin release and testosterone production [9,10]. In humans, mice, and rats, the inhibition of GnRH pulsatility (for instance, using GnRHa) suppresses the hypothalamic–pituitary–gonadal (HPG) axis, leading to the delayed progression of puberty [11,12]. Introduced in the 1980s as the “Dutch protocol” [13], this treatment delays puberty to reduce GD, allowing time for an open exploration of gender identity and facilitating better outcomes for hormonal and surgical treatments. Once of legal age, adolescents may continue, if desired, with gender-affirming hormone therapy (GAHT), using estradiol and cyproterone acetate for transgender girls or testosterone for transgender boys [14].

However, in addition to the known risks of mood swings and emotional lability [15], changes in body composition, slower growth, increased fat, decreased lean mass, reduced bone turnover markers, and lower height are reported in transgender pubertal girls [16,17], and there are two major concerns with GnRHa treatment. First, the unknown effects on brain development, as significant changes in the cortex and white matter during puberty are influenced by sex [18,19,20]. Second, the effects on fertility remain uncertain, which is especially concerning as some transgender children and adolescents may desist from gender incongruence at puberty, while those who persist may wish to preserve their fertility.

Young girls and transgender feminine youths undergoing GAHT for feminization have shown adverse effects on testicular development [21,22]. Estrogen treatment is known to impair spermatogenesis [21,23], reduce the number of testosterone-producing Leydig cells (Schultze, 1984) [24], and replace these cells with fibroblast-like cells [24]. Additionally, it contributes to the development of varicose veins [25] and atherosclerosis [26]. Testicular lesions in transgender women on GAHT depend on the dosage and duration of treatment, ranging from complete spermatogenesis to seminiferous tubules resembling infantile or pubertal stages [21].

As an initial approach, laboratory animal models can be valuable for understanding the effects of GnRHa treatment on the reproductive system. Studies on female mice reported that treatment with the GnRHa goserelin acetate resulted in decreased uterine and ovarian weights and the absence of corpora lutea [27]. Additionally, when female mice were treated with testosterone to induce masculinization, they showed increased clitoral volume, atretic cyst-like late antral follicles, and a continued absence of corpora lutea [28]. There is also wide experience of using GnRHa to induce sterilization in male companion animals [29] and macaques [30]; the sperm quality is reestablished in cats after the cessation of treatment [31]. However, there are no studies on the effects of triptorelin on the testicular tissue during and after the cessation of treatment.

In humans, fertility preservation guidelines have been developed for transgender adults undergoing GAHT [32,33]. However, it remains unclear whether puberty blockade with GnRHa affects the normal structure and function of the testicles in transgender girls. Although it is well known that patients with hypogonadotropic hypogonadism recover testicular function after pulsatile GnRH or gonadotropins treatments [34], no direct studies of the testicular tissue have been undertaken after triptorelin treatment, a GnRHa used to block puberty in transgender girls. Therefore, it is important to assess the condition of the testicles before beginning GAHT with estradiol and an antiandrogen to determine if triptorelin leaves some lesions in the testicular tissue that would be adverse late in life. Since directly assessing testicular status in young transgender girls would require invasive techniques, developing animal models can assist clinicians in advising these individuals on sperm preservation options for future fertility. In this study, we developed an animal model to examine the effects of triptorelin, a GnRHa, on the testicles. We conducted a longitudinal study with three groups of male pubertal rats treated with triptorelin for either 60 or 90 days, along with a control group treated with vehicle for 90 days.

## 2. Results

**Figure 1:**
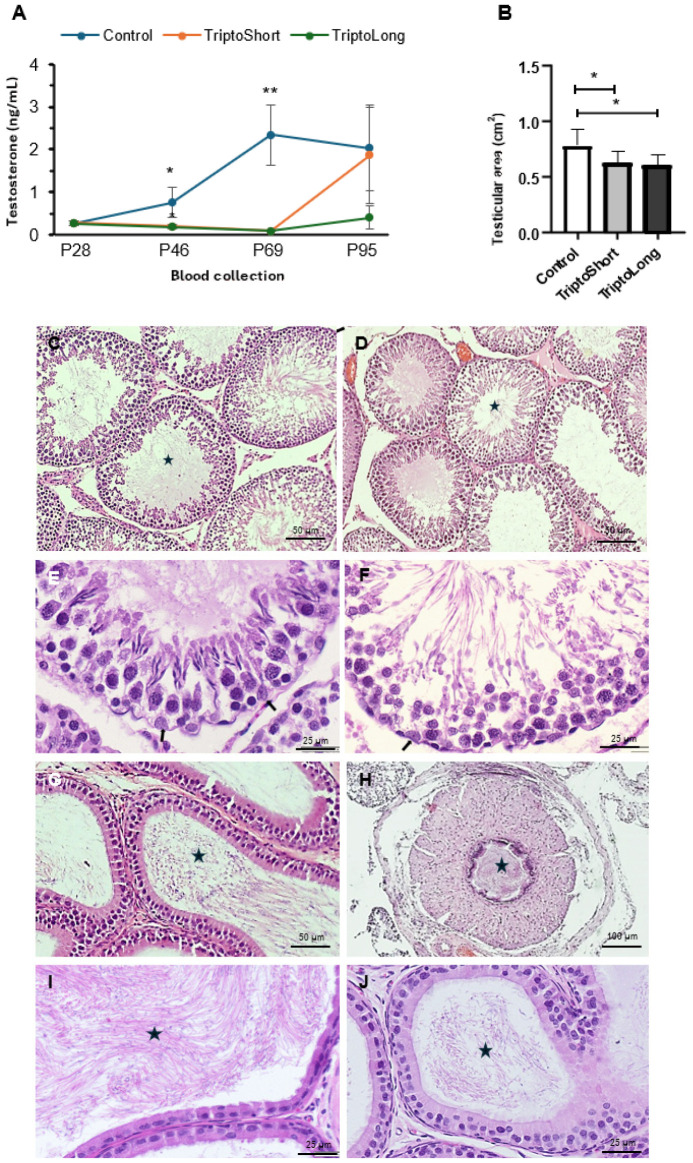
Plasma testosterone and normal histological findings: (**A**): Mean and standard deviation (SEM) of testosterone levels at different time points, demonstrating statistically lower levels in the treated groups compared to controls (* *p* ≤ 0.01; ** *p* ≤ 0.001). (**B**): Mean and SD of the testicular area in the mid-transverse section showing statistical differences at *p* ≤ 0.05 (**C**): Control testes demonstrating several tubules with lumens (stars) with 50 µm complete spermatogenesis and a normal interstitium. (**D**): Typical testicular parenchyma from a rat subjected to short-term treatment, showing several seminiferous tubules with complete spermatogenesis (stars) and a normal interstitium. (**E**): High magnification of a seminiferous tubule of a long-term treated rat showing normal stage XII spermatogenesis, with healthy triangular Sertoli cell nuclei (arrows), and germ cells as zygotene and pachytene spermatocytes and immature spermatids. (**F**): High magnification of a seminiferous tubule of a long-term treated rat showing normal stage VII spermatogenesis, with healthy triangular Sertoli cell nuclei (arrows), and germ cells as preleptotene and pachytene spermatocytes and immature and mature spermatids towards the center of the tubule. (**G**): Section of the epididymis from a short-term treated rat, showing a normal amount of mature sperm (star). (**H**): Section of the vas deferens from a long-term treated rat, also displaying mature sperm (star). (**I**): High magnification of the epididymis of a negative control rat, showing normal morphology of the sperm (star). (**J**): High magnification of the epididymis of a long-term treated rat, showing normal morphology of the sperm (star). Magnification bars: 25, 50, or 100 µm, as indicated.

The first testosterone analysis was conducted on P28, representing early puberty for male Sprague Dawley rats [35], without group distinction. Plasma testosterone values at this stage were consistent with prepubertal levels, at 0.25 ng/mL ± 0.052 (mean ± SEM) (Figure 1A). The second blood collection on P48, corresponding to early pubertal development, showed testosterone levels of 0.76 ng/mL ± 0.35 in controls, aligning with this stage of sexual development, and significantly lower levels of 0.18 ng/mL ± 0.05 in treated rats (*p* = 0.023). By the third collection on P69, the mean testosterone levels in controls had risen to 2.34 ± 0.7 ng/mL, indicating fully established puberty, whereas treated rats maintained low levels at 0.03 ± 0.01 ng/mL (*p* < 0.001).

Although testosterone levels showed high variability in both treated and untreated rats (Figure 1A), a statistically significant reduction in testosterone was observed in triptorelin-treated rats by day P69, indicating a delay in pubertal progression (Figure 1A). By P93, testosterone levels in rats that received vehicle at P71 showed recovery, while levels remained low in the triptorelin-long group (Figure 1A), suggesting a recovery of testicular function and the potential reversibility of triptorelin treatment.

Treated rats generally exhibited smaller testes, with a statistically significant reduction in testicular area compared to the negative-control-vehicle-treated group (Figure 1B). This was observed in both the short-term and long-term treatment groups. Histological examination showed that the majority of the testicular parenchyma in all rats independent of the group was unaffected, mostly containing seminiferous tubules with complete spermatogenesis (Figure 1D) similar to those in negative control rats injected with vehicle (Figure 1C). No changes in the staging of spermatogenesis were observed, as every stage of spermatogenesis was observed in all groups (Figure 1E,F). Similarly, no hypoplasic changes or tumor-related germ cell alterations such as germ cell neoplasia in situ (GCNIS) cells were observed either. The interstitial tissue showed no differences between groups in areas with complete spermatogenesis (compare Figure 1C,D). All treated rats in both groups demonstrated the presence of normal counts of mature sperm of a normal morphology in the epididymis (Figure 1G,J) and vas deferens (Figure 1H), similar to negative control group (Figure 1I), indicating fertility was maintained despite the treatment.

**Figure 2:**
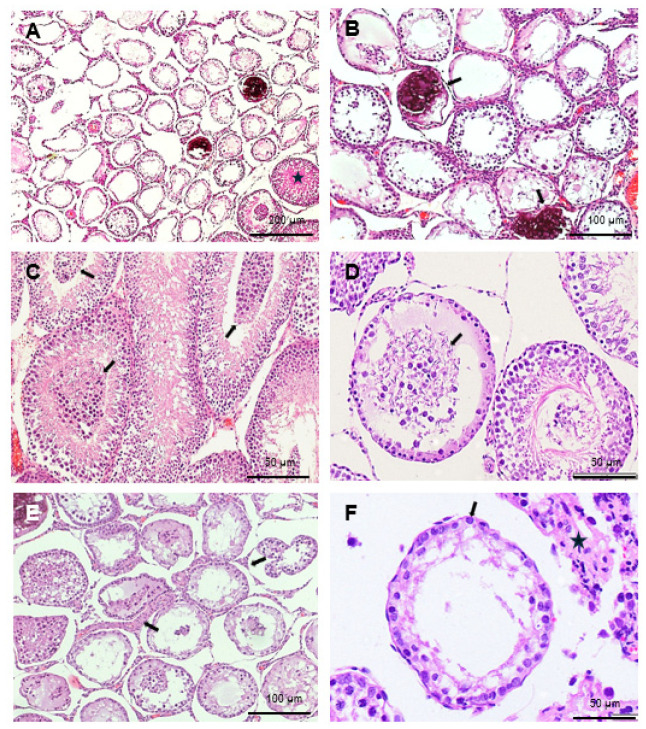
Atrophic histological findings. (**A**): Focal changes in the testes of rats subjected to long-term treatment, exhibiting atrophic tubules with reduced diameter and a lack of advanced spermatogenesis, characterized by cords containing only Sertoli cells and spermatogonia. Note the focal nature of these alterations; a tubule with complete spermatogenesis (star) is also shown in the photograph, while all the atrophic tubules are situated closely together. (**B**): Microlithiasis observed in two seminiferous tubules (arrows) of a long-term treated rat, situated among atrophic tubules. (**C**): Seminiferous tubules with sloughed germ cells in the center (arrows) in a short-term treated rat. (**D**): Seminiferous tubules with sloughed germ cells in the center (arrows) in a long-term treated rat. Note the remaining tubular wall contains only Sertoli cells and spermatogonia. (**E**): Interstitium in a long-term treated rat, showing enlarged interstitial compartments (arrows) in the atrophic area. (**F**): Atrophic tubule in a long-term treated rat, showing Sertoli cells (arrow) and scarce spermatogonia as the only germ cells. The interstitium is enlarged in this area (star).

In addition, discrete and focal atrophic changes, primarily near the rete testis, were noted in both treated groups. These included atrophic tubules with reduced diameter and the absence of advanced spermatogenesis, containing only Sertoli cells (SC) (Sertoli-cell-only (SCO) tubules), or containing only spermatogonia as the germ cell compartment (Figure 2A,B,D–F), as well as signs of microlithiasis (Figure 2A,B,D). A few tubules with sloughed germ cells in the center were also observed (Figure 2C–E), but to a low extent, as they were only occasionally observed in the epididymis, suggesting they were cleared out by Sertoli cells. Atrophic areas in both treated groups also exhibited an enlarged interstitial compartment (Figure 2E,F).

These alterations affected 1–30% of the testicular parenchyma, depending on the individual case, and were classified into two categories: “normal”, where 1–10% of the parenchyma showed lesions (as seen in negative control rats); and “atrophic”, where around 30% of the parenchyma was affected. In the negative control group, all rats were obviously categorized as normal. In the short-term treatment group, approximately two-thirds of the rats were classified as normal, while one-third (29%) were categorized as atrophic. In the long-term treatment group, 50% were classified as normal, and 50% as atrophic. These findings correlate with the high standard deviation observed in testosterone levels across the groups (Figure 1A).

## 3. Discussion

In this study, triptorelin treatment was initiated on day 30, prior to the onset of puberty in rats (day 40), to mimic the timing of GnRH agonist treatment in transgender girls. The treatment was concluded on day 95, which corresponds to adulthood in humans [35]. This timing was chosen to mimic the treatment in patients, usually starting before puberty and finishing with the achievement of adulthood, when either gender-affirming hormone therapy is elected and started, or the treatment is abandoned because they desist from gender incongruence.

We observed a drop in testosterone levels accompanied by discrete focal atrophic testicular lesions, which were partially reversible and compatible with sperm production and fertility preservation in both treated groups.

Previous morphological studies have described testicular changes in transgender women due to estrogen exposure [24,36,37,38,39]. These changes are characterized by the absence of true spermatogenesis in the seminiferous tubules, which instead contain seminiferous cords with no lumen, comprising Sertoli cells (SCs) and spermatogonia as the only germ cells. These cords are surrounded by a thickened lamina propria and an enlarged interstitial compartment that contains fibroblast-like cells rather than androgen-producing Leydig cells (LCs). In previous studies conducted by our group [40], we identified inhibin bodies, a new marker for immature SCs, in the apical cytoplasm of hypoplastic tubules in the two cases of gender transition we studied. This suggests that antiandrogen and estrogen exposure in the adult human testis induces changes in SCs that do not merely represent the atrophy of the seminiferous tubule.

In a larger study involving 11 individuals undergoing elective gender transition with varying doses and durations of estrogen treatment [21], our findings demonstrated diffuse and true SC dedifferentiation marked by the re-expression of immature SC markers (M2A antigen, inhibin bodies, and Anti-Müllerian Hormone), along with the absence of the SC maturation marker androgen receptor in involuting seminiferous tubules, highlighting the plasticity of adult human SCs. The observed changes were dose-dependent and suggested true testicular dedifferentiation induced by estrogens. Despite these lesions, complete spermatogenesis was also noted in some tubules in our studies [21,22] and confirmed by others [41,42,43].

In contrast to the effects of estrogen treatment, GnRH agonist treatment in this study induced histological changes that were focal rather than diffuse. Spermatogenesis was maintained and normal in the majority of the testicular parenchyma, and sperm amount and morphology were also similar to the negative control group, ensuring the maintenance of fertility despite the treatment. Additionally, no SC dedifferentiation was observed; however, in the treated groups, up to 30% atrophy of seminiferous tubules in the testicular parenchyma was noted in some of the rats, demonstrating a clear difference in the mechanisms of action and effects on the testicular tissue between GnRH agonists and estrogen treatment. In the rats, spermatogonia were already present at 2 days port-partum (dpp); spermatocytes appeared around 15 dpp, corresponding to 7–12 years in humans; and spermatids at 30 dpp (corresponding to prepubertal age 14–16 years in humans). That means that when triptorelin was administered at 30 dpp, both spermatocytes and spermatids were already present in all seminiferous tubules of the rat testes. Hence, the presence of seminiferous tubules with Sertoli cells and spermatogonia only clearly implicates that the rest of the more advanced germ cells will disappear. This also correlates with old studies showing that both spermatids and spermatocytes are the first germ cells to degenerate [44]. This further correlates with the presence of sloughed germ cells in the seminiferous tubule lumen of treated rats (Figure 2C,D). In addition, hypoplasic tubules would show an inexistent or very narrow tubular lumen, as it does not develop until spermatocytes progress. In our case (Figure 2D,F) it can be determined that the lumen is of a similar diameter to normal spermatogenesis tubules both in the untreated and treated groups (Figure 1C,D), further suggesting that atrophy has occurred.

Our results concur with data from GnRH-deficient patients, such as those suffering from Kallman syndrome, a genetic form of hypogonadotropic hypogonadism disorder caused by mutations in the *KAL1* gene characterized by delayed or absent puberty. A review of the recent literature reveals numerous clinical manifestations in these patients, including microgenitalia, impotence, reduced libido, and infertility (Yadav et al., 2025) [45]. In these patients, the treatment goal is the development of secondary sexual characteristics, and the restoration of fertility with pulsatile GnRH therapy and also with gonadotropin therapy (either human chorionic gonadotropin *(*HCG) alone or combined with follicle-stimulating hormone (FSH)) [46,47,48]. However, for obvious reasons, no data on testicular histology is available. The dose and duration of our treatments, even in the long-term group, were obviously lower than for this type of patient, as they were designed to mimic treatment in prepubertal male patients treated with triptorelin, which explains the lower affectation of the testicular parenchyma and the maintenance of sperm production and thus fertility.

Our study has at least one limitation: we observed high variability in serum testosterone levels as well as histological findings. Despite this variability, a statistically significant reduction in testosterone levels on day P69 was noted in triptorelin-treated rats, indicating a delay in puberty. By P93, testosterone levels had recovered in short-term treated rats, while they remained low in the long-term triptorelin group, suggesting a potential recovery of testicular function and the reversibility of triptorelin treatment. This aligns with previous data from Han et al. [49], which also reported a recovery of testosterone following the cessation of triptorelin treatment in rats. The high variability in testosterone levels showed poor correlation with histological parameters, possibly due to the relatively short duration of treatments, as histological changes typically follow a drop in testosterone levels. Longer treatment durations might yield more homogeneous histological and testosterone measurements in treated rats. Alternatively, increasing the number of animals per group could provide more reliable results, as even the control group exhibited high variability, likely due to the prepubertal timing of treatment initiation, which varied among individuals.

In our study, we found that treated animals exhibited reduced testicular size, consistent with previous findings in prepubertal male patients treated with triptorelin [13].

Focal atrophy of the seminiferous tubules was predominantly observed near the rete testis, even though triptorelin was administered intramuscularly. Both Matoso et al. [42] and Peña-Barreno et al. [22] reported that patients treated with estradiol for gender reassignment did not show alterations in the rete testis, but instead exhibited epididymal epithelial hyperplasia and periepididymal fibrosis. In our study involving triptorelin, we observed no such alterations or any others in the epididymis, which can be attributed to the differences in treatment.

We noted variability in the number of rats exhibiting more extensive lesions, with only one-third of the short-term treated rats showing atrophy compared to half of those in the long-term treatment group. This suggests a recovery of the testicular parenchyma after treatment cessation. It is noteworthy that the differences are encountered in the number of animals showing such lesions, but not in the amount of testicular parenchyma affected, which is never higher than 30%, never diffuse, never causes cancer-related alterations (no germ cell neoplasia in situ (GCNIS) was observed at all), and never causes infertility.

Crucially, testicular sperm production and travel to the epididymis, and hence fertility, were preserved in all treated rats. This would be highly beneficial for patients, ensuring fertility preservation through non-invasive sperm cryopreservation prior to the initiation of estrogen treatment [33].

In conclusion, our data suggests that triptorelin treatment leads to a decline in testosterone levels accompanied by the discrete atrophy of the seminiferous tubules, which is partially reversible and compatible with sperm production and fertility preservation. This indicates that triptorelin could be an appropriate treatment prior to estrogen therapy for patients seeking gender transition.

## 4. Materials and Methods

### 4.1. Subjects

Thirty 23-day-old male Sprague Dawley rats (Janvier Labs, Le Genest St. Isle, France) were housed and handled in the animal facility at the Instituto de Investigaciones Biomédicas Sols-Morreale (IIBM) in Madrid, Spain, in compliance with European Union standards for laboratory animal care (2010/63/EU). The study was conducted according to the guidelines of the Declaration of Helsinki and all animal procedures were approved by the Institutional Review Board of the IIBM-CSIC Ethics Committee on Animal Experimentation and the Dirección General de Agricultura, Ganadería y Alimentación of the Comunidad de Madrid (Autonomous Community of Madrid Directorate General for Agriculture, Livestock and Food) (protocol code PROEX 126.2/23). The rats were housed three per cage in a room maintained at a constant temperature of 22 ± 2 °C, 40% humidity, and a 12 h light/dark cycle, with food and water provided ad libitum.

### 4.2. Experimental Design

**Figure 3:**
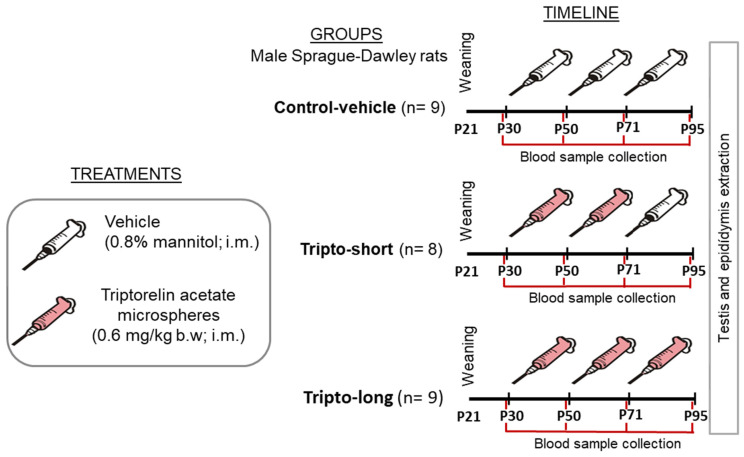
Experimental design: Includes the timeline and ages for each triptorelin or vehicle injection (postnatal days 30, 50, and 71). Blood samples were collected from the tail vein two days before each treatment and two days before sacrifice (P93). Testes and epididymides were collected at the time of sacrifice on P95.

The experimental design is outlined in Figure 3. The rats were randomly assigned to one of three groups: negative control (injected with vehicle), triptorelin-short, and triptorelin-long groups, with each group comprising 10 animals. Treatments were administered intramuscularly under isofluorane anesthesia (2% induction, 4% maintenance). Rats in the triptorelin-short group received triptorelin (Decapeptyl depot, Ipsen Pharma, Paris, France) at a dose of 0.6 mg/kg body weight on postnatal days (P) 30 and 50, followed by only vehicle on P71. The triptorelin-long group received the same triptorelin dose at all three time points (P30, P50, and P71). Control-vehicle-group rats received only vehicle, which was the solvent for the triptorelin preparation, at the same time points. On P95, following euthanasia by sodium pentobarbital overdose, the testes and epididymides were removed and fixed in 10% neutral-buffered formalin.

### 4.3. Blood Collection and Testosterone Quantification

Blood samples were collected in heparinized tubes (Microvette^®^ 300, Sarstedt, Germany) from the tail vein two days before each triptorelin administration (P28, P48, and P69) and two days before sacrifice (P93). Plasma was obtained by centrifuging the blood samples at 2000× *g* for 5 min at 20 °C and then stored at −80 °C until analysis.

Plasma testosterone levels were measured using quantitative multiple reaction monitoring (MRM) analysis (LC-QQQ-MS) with a Shimadzu LC-MS8030 system (Shimadzu, Kyoto, Japan) and a Phenomenex Gemini 5u C18 110 A 150 × 2 mm column. Briefly, 40 μL of serum was mixed with 200 μL of methanol for protein precipitation. After 1 min of vortex mixing, samples were centrifuged at 14,000× *g* for 10 min at 4 °C. A 200 μL aliquot of each sample was dried using a speed-vac and reconstituted in 100 μL of methanol. Samples were then filtered through a 0.22 μm PTFE filter and analyzed.

### 4.4. Histology

The testes and epididymides were routinely fixed in formalin and embedded in paraffin for histological examination. Transverse sections, 5 μm thick, were stained with H&E, and the testicular area was measured as an indicator of testicular size. The testicular and epididymal parenchyma were examined histologically and photographed using a Zeiss microscope (Carl Zeiss AG, Oberkochen, Germany). Seminiferous tubules were staged, and germ cells’ morphology was studied (presence of germ cell neoplasia in situ (GCNIS), slaughtered germ cells, arrest of spermatogenesis) as well as other signs of hypoplasia or atrophy (presence of Sertoli-cell-only tubules, microlithiasis), Sertoli cell morphology (triangular or round nucleus, height of cell, presence of Sertoli cell–germ cell junctions), and interstitial Leydig cell count and morphology. Epididymis and vas deferens were also examined both in terms of epithelial changes (at the three portions of epididymis caput, corpus, and cauda) and sperm content and morphology were also examined.

### 4.5. Statistics

A Mann–Whitney U test was used to compare the blood testosterone concentration at P48 and P69 between treated (triptorelin-short and triptorelin-long) and untreated (control) subjects. The Kruskal–Wallis test was used to assess testosterone concentration group differences at P93. Testicular area significance was assessed by the Student t test. All statistical analyses were performed using SPSS version 29, with significance set at a minimum of *p* ≤ 0.05.

## Data Availability

The data underlying this article is available in the article.

## References

[1] Stoller R. (1964). A Contribution to the Study of Gender Identity. Int. J. Psychoanal..

[2] VandenBos G.R. (2015). APA Dictionary of Psychology.

[3] American Psychiatric Association (2013). Diagnostic and Statistical Manual of Mental Disorders.

[4] Herman J.L., Flores A.R., O’Neill K.K. (2022). How Many Adults and Youth Identify as Transgender in the United States?. https://williamsinstitute.law.ucla.edu/publications/trans-adults-united-states/.

[5] Arcelus J., Bouman W.P., Van Den Noortgate W., Claes L., Witcomb G., Fernandez-Aranda F. (2015). Systematic Review and Meta-Analysis of Prevalence Studies in Transsexualism. Eur. Psychiatry.

[6] Zhang Q., Goodman M., Adams N., Corneil T., Hashemi L., Kreukels B., Motmans J., Snyder R., Coleman E. (2020). Epidemiological Considerations in Transgender Health: A Systematic Review with Focus on Higher Quality Data. Int. J. Transgend Health.

[7] American Psychiatric Association (2022). Diagnostic and Statistical Manual of Mental Disorders: Text Revision.

[8] World Professional Association for Transgender Health (WPATH) (2022). Standards of Care for the Health of Transgender and Gender Diverse People, Version 8.

[9] Kiesel L.A., Rody A., Greb R.R., Szilágyi A. (2002). Clinical Use of GnRH Analogues. Clin. Endocrinol..

[10] Ortmann O., Weiss J.M., Diedrich K. (2002). Gonadotrophin-Releasing Hormone (GnRH) and GnRH Agonists: Mechanisms of Action. Reprod. Biomed. Online.

[11] Dipalma J.R. (1990). Tartrazine Sensitivity. Am. Fam. Physician.

[12] Gill J.C., Wang O., Kakar S., Martinelli E., Carroll R.S., Kaiser U.B. (2010). Reproductive Hormone-Dependent and -Independent Contributions to Developmental Changes in Kisspeptin in GnRH-Deficient Hypogonadal Mice. PLoS ONE.

[13] Delemarre-Van De Waal H.A., Cohen-Kettenis P.T. (2006). Clinical Management of Gender Identity Disorder in Adolescents: A Protocol on Psychological and Paediatric Endocrinology Aspects. Eur. J. Endocrinol..

[14] Schagen S.E.E., Cohen-Kettenis P.T., Delemarre-van de Waal H.A., Hannema S.E. (2016). Efficacy and Safety of Gonadotropin-Releasing Hormone Agonist Treatment to Suppress Puberty in Gender Dysphoric Adolescents. J. Sex. Med..

[15] Khatchadourian K., Amed S., Metzger D.L. (2014). Clinical Management of Youth with Gender Dysphoria in Vancouver. J. Pediatr..

[16] Rew L., Young C.C., Monge M., Bogucka R. (2021). Review: Puberty Blockers for Transgender and Gender Diverse Youth—A Critical Review of the Literature. Child. Adolesc. Ment. Health.

[17] Ramos G.G.F., Mengai A.C.S., Daltro C.A.T., Cutrim P.T., Zlotnik E., Beck A.P.A. (2021). Systematic Review: Puberty Suppression with GnRH Analogues in Adolescents with Gender Incongruity. J. Endocrinol. Investig..

[18] Puy L., MacLusky N.J., Becker L., Karsan N., Trachtenberg J., Brown T.J. (1995). Immunocytochemical Detection of Androgen Receptor in Human Temporal Cortex: Characterization and Application of Polyclonal Androgen Receptor Antibodies in Frozen and Paraffin-Embedded Tissues. J. Steroid Biochem. Mol. Biol..

[19] Raznahan A., Lee Y., Stidd R., Long R., Greenstein D., Clasen L., Addington A., Gogtay N., Rapoport J.L., Giedd J.N. (2010). Longitudinally Mapping the Influence of Sex and Androgen Signaling on the Dynamics of Human Cortical Maturation in Adolescence. Proc. Natl. Acad. Sci. USA.

[20] Österlund M.K., Gustafsson J., Keller E., Hurd Y.L. (2000). Estrogen Receptor Beta (ERβ) Messenger Ribonucleic Acid (MRNA) Expression within the Human Forebrain: Distinct Distribution Pattern to ERα MRNA. J. Clin. Endocrinol. Metab..

[21] Nistal M., Gonzalez-Peramato P., De Miguel M.P. (2013). Sertoli Cell Dedifferentiation in Human Cryptorchidism and Gender Reassignment Shows Similarities between Fetal Environmental and Adult Medical Treatment Estrogen and Antiandrogen Exposure. Reprod. Toxicol..

[22] Peña Barreno C., Gonzalez-Peramato P., Nistal M. (2020). Vascular and Inflammatory Effects of Estrogen and Anti-Androgen Therapy in the Testis and Epididymis of Male to Female Transgender Adults. Reprod. Toxicol..

[23] Cheng P.J., Pastuszak A.W., Myers J.B., Goodwin I.A., Hotaling J.M. (2019). Fertility Concerns of the Transgender Patient. Transl. Androl. Urol..

[24] Schulze C. (1988). Response of the Human Testis to Long-Term Estrogen Treatment: Morphology of Sertoli Cells, Leydig Cells and Spermatogonial Stem Cells. Cell Tissue Res..

[25] Kendler M., Blendinger C., Haas E. (2009). Elevated Serum Estradiol/Testosterone Ratio in Men with Primary Varicose Veins Compared with a Healthy Control Group. Angiology.

[26] Murakami H., Harada N., Sasano H. (2001). Aromatase in Atherosclerotic Lesions of Human Aorta. J. Steroid Biochem. Mol. Biol..

[27] Dela Cruz C., Kinnear H.M., Hashim P.H., Wandoff A., Nimmagadda L., Chang A.L., Padmanabhan V., Shikanov A., Moravek M.B. (2023). A Mouse Model Mimicking Gender-Affirming Treatment with Pubertal Suppression Followed by Testosterone in Transmasculine Youth. Hum. Reprod..

[28] Kinnear H.M., Constance E.S., David A., Marsh E.E., Padmanabhan V., Shikanov A., Moravek M.B. (2019). A Mouse Model to Investigate the Impact of Testosterone Therapy on Reproduction in Transgender Men. Hum. Reprod..

[29] Lucas X. (2014). Clinical Use of Deslorelin (GnRH Agonist) in Companion Animals: A Review. Reprod. Domest. Anim..

[30] Tran H.D., Carroll K.E., Mackiewicz A.L., Ardeshir A., Stockinger D., De Lucena T., Christe K.L. (2023). Effects of Deslorelin on Testosterone Secretion and Testicular Volume in Male Rhesus Macaques (Macaca Mulatta). J. Am. Assoc. Lab. Anim. Sci..

[31] Nuñez Favre R., García M.F., García Mitacek M.C., Rearte R., Fontaine C., de la Sota R.L., Stornelli M.A. (2018). Reestablishment of Sperm Quality after Long-Term Deslorelin Suppression in Tomcats. Anim. Reprod. Sci..

[32] Anderson R.A., Amant F., Braat D., D’Angelo A., De Sousa Lopes S.M.C., Demeestere I., Dwek S., Frith L., Lambertini M., Maslin C. (2021). ESHRE Guideline: Female Fertility Preservation. Hum. Reprod. Open.

[33] Henry L., Berek J.S., Diaz I., Feldberg D., Mocanu E., Niederberger C.C., Ohlander S., Purandare N., Rosenwaks Z., Tulandi T. (2023). FIGO Statement: Fertility Preservation. Int. J. Gynecol. Obstet..

[34] Boehm U., Bouloux P.M., Dattani M.T., De Roux N., Dodé C., Dunkel L., Dwyer A.A., Giacobini P., Hardelin J.P., Juul A. (2015). Expert Consensus Document: European Consensus Statement on Congenital Hypogonadotropic Hypogonadism-Pathogenesis, Diagnosis and Treatment. Nat. Rev. Endocrinol..

[35] Ghasemi A., Jeddi S., Kashfi K. (2021). The Laboratory Rat: Age and Body Weight Matter. EXCLI J..

[36] de la Baize F.A., Mancini R.E., Bur G.E., Irazu J. (1954). Morphologic and Histochemical Changes Produced by Estrogens on Adult Human Testes. Fertil. Steril..

[37] Lu C.C., Steinberger A. (1978). Effects of Estrogen on Human Seminiferous Tubules: Light and Electron Microscopic Analysis. Am. J. Anat..

[38] PAYER A.F., MEYER W.J., WALKER P.A. (1979). The Ultrastructural Response of Human Leydig Cells to Exogenous Estrogens. Andrologia.

[39] Schulze C. (1984). Sertoli Cells and Leydig Cells in Man.

[40] Nistal M., Pastrián L.G., González-Peramato P., De Miguel M.P. (2011). Inhibin Bodies: A New Marker for Immature Sertoli Cells. Histopathology.

[41] Schneider F., Neuhaus N., Wistuba J., Zitzmann M., Heß J., Mahler D., van Ahlen H., Schlatt S., Kliesch S. (2015). Testicular Functions and Clinical Characterization of Patients with Gender Dysphoria (GD) Undergoing Sex Reassignment Surgery (SRS). J. Sex. Med..

[42] Matoso A., Khandakar B., Yuan S., Wu T., Wang L.J., Lombardo K.A., Mangray S., Mannan A.A.S.R., Yakirevich E. (2018). Spectrum of Findings in Orchiectomy Specimens of Persons Undergoing Gender Confirmation Surgery. Hum. Pathol..

[43] Kent M.A., Winoker J.S., Grotas A.B. (2018). Effects of Feminizing Hormones on Sperm Production and Malignant Changes: Microscopic Examination of Post Orchiectomy Specimens in Transwomen. Urology.

[44] Russell L.D., Ettlin R.A., Sinha Hikim A.P., Clegg E.D. (1990). Histological and Histopathological Evaluation of the Testis.

[45] Kumar Yadav R., Qi B., Wen J., Gang X., Banerjee S. (2025). Kallmann Syndrome: Diagnostics and Management. Clin. Chim. Acta.

[46] Dwyer A.A., Sykiotis G.P., Hayes F.J., Boepple P.A., Lee H., Loughlin K.R., Dym M., Sluss P.M., Crowley W.F., Pitteloud N. (2013). Trial of Recombinant Follicle-Stimulating Hormone Pretreatment for GnRH-Induced Fertility in Patients with Congenital Hypogonadotropic Hypogonadism. J. Clin. Endocrinol. Metab..

[47] Zheng J., Mao J., Xu H., Wang X., Huang B., Liu Z., Cui M., Xiong S., Ma W., Min L. (2017). Pulsatile GnRH Therapy May Restore Hypothalamus-Pituitary-Testis Axis Function in Patients with Congenital Combined Pituitary Hormone Deficiency: A Prospective, Self-Controlled Trial. J. Clin. Endocrinol. Metab..

[48] Grob F., Keshwani R., Angley E., Zacharin M. (2024). Fertility Outcomes in Male Adults with Congenital Hypogonadotropic Hypogonadism Treated during Puberty with Human Chorionic Gonadotropin and Recombinant Follicle Stimulating Hormone. J. Paediatr. Child. Health.

[49] Han J., Zhang S., Liu W., Leng G., Sun K., Li Y., Di X. (2014). An Analytical Strategy to Characterize the Pharmacokinetics and Pharmacodynamics of Triptorelin in Rats Based on Simultaneous LC-MS/MS Analysis of Triptorelin and Endogenous Testosterone in Rat Plasma. Anal. Bioanal. Chem..

